# Role of ecto-5’-nucleotidase in bladder function activity and smooth muscle contractility

**DOI:** 10.1007/s11302-024-10015-0

**Published:** 2024-05-08

**Authors:** Basu Chakrabarty, Bahareh Vahabi

**Affiliations:** 1 School of Physiology, Pharmacology & Neuroscience, Faculty of Health & Life Sciences, University of Bristol, Bristol, UK; 2 School of Applied Sciences, University of West England, Bristol, UK

**Keywords:** ATP, Bladder smooth muscle, Ecto-5’-nucleotidase, Purinergic signalling

## Article Summary

The purinergic system regulates bladder function through the action of ATP and its active metabolites. This signalling mechanism involves ATP, ADP and adenosine binding to specific receptors, including purine-gated cation channels (P2X_1-7_) and various G protein coupled receptors (nucleotides – P2Y_1,2,4,6,11–14_ and adenosine – A_1_, A_2A_, A_2B_, A_3_). The breakdown of purines is facilitated by enzymes, such as ectonucleoside triphosphate diphosphohydrolases (ENTPD1, 2, 3, 8), which convert ATP into ADP and then to AMP, and by ecto-5’-nucleotidase (NT5E) and urothelial alkaline phosphatase (ALPL), which convert AMP into adenosine. Dysfunction in ATP breakdown and signalling are linked to lower urinary tract symptoms in patients, affecting the contractility of detrusor smooth muscle through altered purinergic mechanisms [1, 2].

Previous animal studies suggest that the interplay between adenosine A_2B_ receptors and nucleotide P2Y_12_ receptors, via ADP action, regulates bladder contractility. Knockout mice lacking *P2y12* or *Adora2b* (A2bKO) gene expression exhibit opposing effects on bladder function parameters [3, 4]. *Entpd1* knockout mice also show dysregulated bladder smooth muscle contractility due to abnormal purine kinetics, whilst bladders from *Nt5e* knockout (Nt5eKO) mice demonstrate altered responses to ADP stimulation [4].

In a recent study, Barge et al., (2024) investigated the role of NT5E in modulating detrusor smooth muscle contractility using Nt5eKO mice [5]. Various assays were used to confirm the absence of NT5E expression in mouse bladders, and bladder function and contractility were assessed. Nt5eKO mice displayed increased voiding frequency with reduced voiding intervals and decreased bladder compliance. This study highlights the involvement of NT5E in maintaining bladder function by modulating smooth muscle activity. The bladder abnormalities in Nt5eKO mice were milder than those seen in A_2B_KO mice and it has been proposed that the absence of NT5E might trigger compensatory mechanisms, including P2Y_12_ receptor downregulation and ALPL upregulation, to mitigate voiding dysfunction [3–5].

## Commentary

The purinergic signalling system plays a diverse role in regulating both sensory and motor functions of the bladder. Changes in purinergic sensory and motor pathways are implicated in bladder dysfunction, and if these pathways can be targeted, it may provide potential novel drug targets. Previous studies in animals have elucidated the involvement of various purines and their receptors in bladder function modulation [3, 4]. ADP-induced detrusor muscle contraction is blocked by a selective P2Y_12_ receptor antagonist. After an immediate contraction initiated by ADP, prolonged exposure causes detrusor muscle to become refractory to further ADP-mediated contraction. However, this response is altered in *Entpd1* or Nt5eKO mice, or upon inhibition of adenosine signalling, suggesting that ADP-P2Y_12_ signalling is regulated by ENTPD1/NT5E activity and adenosine receptors [4]. Adenosine acting at A_2B_ receptors, inhibited purinergic detrusor contractions. *P2y12* knockout and A_2B_KO mice had contrasting phenotypes, with *P2y12* knockout mice exhibiting an underactive bladder phenotype with increased bladder capacity and reduced voiding frequency, whereas A_2B_KO mice had an overactive bladder, with decreased bladder capacity and increased voiding frequency. It has been suggested that the interplay of A_2B_ and P2Y_12_ receptors through modulation of adenylyl cyclase-cAMP signalling can regulate bladder contractility [3]. The conversion of ADP/AMP to adenosine by ENTPD1 and NT5E may have contributed to this receptor crosstalk [4]. NT5E catalyses the conversion of extracellular AMP to adenosine and the focus of this study was to test if modulating NT5E activity affects bladder smooth muscle activity.

To study the effect of NT5E, the authors used Nt5eKO mice and confirmed the loss of NT5E expression in this animal model by using quantitative RT-PCR to demonstrate a reduction in *Nt5e* mRNA abundance and Western blotting showed a complete disappearance of a ~ 75 kDa polypeptide in lysate samples from Nt5eKO bladders. Immunofluorescence staining corroborated the absence of NT5E in Nt5eKO mice, which was localised in the detrusor muscle of wild-type mice. Nucleotidase histochemistry was used to determine bladder tissue activities of ATPase, ADPase, and AMPase. Detrusor smooth muscle displayed decreased AMP hydrolysis in Nt5eKO mice compared to wild-type, with no effects on AMP hydrolysis in the lamina propria and urothelium. Interestingly, ATP hydrolysis activity in the lamina propria of Nt5eKO mice increased, suggesting compensatory ENTPD2 upregulation. There were no differences in ADP hydrolysis activity between Nt5eKO and wild-type mice [5].

To determine the voiding phenotype of Nt5eKO mice, voiding spot assays were performed on male and female mice. Nt5eKO male and female mice had more primary voiding spots (PVS) and reduced average PVS area, although the total voided volume between wild-type and Nt5eKO was the same. These results suggest an increased void frequency and smaller void volume in Nt5eKO mice, irrespective of sex. Similar alterations in the voiding phenotype were reflected in functional urodynamic studies using cystometry in anaesthetised mice. Nt5eKO mice had a reduced voiding interval in comparison to wild-type mice, with no differences in basal and peak pressures measured. Bladder compliance, the volume in µl required to increase pressure by one cm H_2_O, was reduced in Nt5eKO mice [5].

Bladder smooth muscle contractility was studied using myography. Electrical field stimulation resulted in frequency-dependent contractions of detrusor smooth muscle, which were reduced in Nt5eKO mice when compared to wild-type. High-K^+^ resulted in a contraction by directly depolarising the smooth muscle, which was also reduced in Nt5eKO mice. This suggests that reduced contractile force in Nt5eKO mice may be intrinsic to effects directly on detrusor smooth muscle rather than the release of neurotransmitters from the efferent nerves innervating detrusor muscle. Atropine-resistant, purinergic neurogenic contractions were reduced in Nt5eKO mice across the stimulation frequency range when compared to wild-type animals. The reduction in nerve-mediated contractions in the presence of α,βme-ATP (10 µM) in Nt5eKO mice also suggests an effect on the cholinergic component of nerve-mediated contractions [5].

Western blots and quantitative RT-PCR demonstrated that mRNA and protein expression levels were similar for both muscarinic M3 receptors and purinergic P2X1 receptors in Nt5eKO and wild-type mice. ALPL is expressed in urothelial cells, and there was an upregulation in its mRNA and protein expression in Nt5eKO mice. ENTPD2, an ATPase expressed in interstitial cells, was upregulated and there was also an increase in ATP hydrolysis in the lamina propria in Nt5eKO mice when compared to wild-type. ENTPD1, which is expressed on the cell surface of detrusor smooth muscle was also upregulated in Nt5eKO mice. Immunoblot studies of P2Y_12_ and A_2B_ receptors were not possible in Nt5eKO mice as there were no appropriate immunospecific antibodies, however, quantitative RT-PCR showed that mRNA expression of P2Y_12_ receptors was reduced and that of A_2B_ receptors was unchanged in Nt5eKO mice bladders when compared to wild-type [5]. The authors highlight that the abnormal bladder phenotype of Nt5eKO mice is much milder than previously reported in A_2B_KO mice. This may be due to a compensatory mechanism, with the reduction of P2Y_12_ receptor protein expression as a negative feedback response to elevated ADP signalling. A_2B_ receptor expression remaining unchanged in Nt5eKO mice suggests a shift in the balance of P2Y_12_-A_2B_ receptor signalling towards A_2B_ receptor [3]. The upregulation of ALPL could potentially offset the voiding dysfunction caused by the absence of NT5E [5].

This study demonstrates that NT5E activity has an effect on bladder activity, with alterations in AMP hydrolysis, the voiding phenotype, functional urodynamics and smooth muscle contractility in Nt5eKO mice, when compared to wild-type [5]. The compensatory changes in other targets involved in the hydrolysis of purines implicated in Nt5eKO mice are of interest. A limitation of this study is that myography experiments were undertaken in isolated detrusor muscle preparations from the bladder and did not look at stretch-induced urothelial ATP release, hence any paracrine mechanism involving urothelial ATP release is inferred and a comparison of myography studies in urothelium-intact versus urothelium-denuded bladder tissue preparations may be valuable. Further studies are required to understand the impact of NT5E activity on urothelial-released ATP and its metabolites and downstream implications on purinergic sensory signalling pathways and interplay with the modulation of bladder muscle function. This study further highlights the intricate purinergic signalling network in various tissues across the bladder, which includes compensatory mechanisms in response to disruptions. These findings contribute to our understanding of bladder pathophysiology and the potential for therapeutic interventions targeting purinergic signalling in the bladder.

## Data Availability

No datasets were generated or analysed during the current study.
